# Microstructure and Properties of Electroless Ni-P/Si_3_N_4_ Nanocomposite Coatings Deposited on the AW-7075 Aluminum Alloy

**DOI:** 10.3390/ma14164487

**Published:** 2021-08-10

**Authors:** Kazimierz Czapczyk, Paweł Zawadzki, Natalia Wierzbicka, Rafał Talar

**Affiliations:** 1 Faculty of Ocean Engineering and Ship Technology, Gdańsk University of Technology, G. Narutowicza 11/12, 80-233 Gdansk, Poland; kazimierz.czapczyk@pg.edu.pl; 2 Faculty of Mechanical Engineering, Poznan University of Technology, Piotrowo 3, 60-965 Poznan, Poland; natalia.wierzbicka@put.poznan.pl (N.W.); rafal.talar@put.poznan.pl (R.T.)

**Keywords:** aluminum alloy, nickel, nanocomposite layers, tribology

## Abstract

The article presents the results of mechanical and tribological tests of Ni-P/Si_3_N_4_ nanocomposite coatings deposited on the AW-7075 aluminum alloy using the chemical reduction method. The influence of the chemical composition on the Vickers microhardness determined by the DSI method was examined. The nanocomposite layers were made of Si_3_N_4_ silicon nitride in a polydisperse powder with a particle size ranging from 20 to 25 nm. The influence of the content of the dispersion layer material on the adhesion to the substrate was analyzed. The abrasive wear was tested and determined in the reciprocating motion using the “ball-on-flat” method. The surface topography was examined by the contact method with the use of a profilometer. Based on the obtained test results, it was found that the Ni-P/Si_3_N_4_ layers produced in the bath with the Si_3_N_4_ nanoparticle content in the amount of 2 g/dm^3^ are more resistant to wear and show greater adhesion than the Ni-P/Si_3_N_4_ layers deposited in the bath with 5 g/dm^3^ of the dispersion phase. NiP/Si_3_N_4_ layers provide protection against abrasive wear under various loads and environmental conditions.

## 1. Introduction

AW-7075 aluminum alloy is an important construction material in any industry because of its lighter weight and better corrosion resistance compared to steel. Some aluminum alloys have similar mechanical properties to structural steels, such as C45 or C46. In this case, the AW-7075 alloy is often used in the shipbuilding, automotive, railway and especially aviation industries [1]. On the other hand, aluminum alloys are less resistant to abrasive wear than steel parts. Increasing the tribological properties can be achieved through an anti-wear layer; however, the appropriate matching of the coating and the substrate depends on their mutual synergism. It is an essential element that determines the proper cooperation of the deposited layer with the aluminum core in selected operating conditions. Aluminum alloys can be coated with various coatings for technical applications [2]. Regarding the mutual antagonism of the substrate and the layer, special attention should be paid to maintaining the basic tribological properties, due to the possibility of chipping, cracking, etc. Mohammadi et al. [3] fabricated Al_2_O_3_/Si_3_N_4_ nanocomposite coating on the surface of the commercial aluminum alloy. The results showed that the hardness of coatings increased from 380 ± 50 HV for the anodized layer to up to 712 ± 36 HV for the composite coating. Moreover, the mass loss decreased from 0.35 for the anodized coating to 0.20 for the coating with Si_3_N_4_ nanoparticles. Researches focused on the optical and mechanical properties of spinel/Si_3_N_4_ nanocomposite with different contents of Si_3_N_4_ were conducted by Nassajpour-Esfahani et al. [4]; in their conclusions, they noted that an increase in the Si_3_N_4_ content is associated with an increase in hardness. However, the highest fracture toughness is achieved in the sample with 1 wt% Si_3_N_4_ content. Further addition dramatically decreases the fracture toughness, caused by an increase in the volume fraction of porosities.

Currently, nanocomposite coatings are of great interest. The use of aluminum alloys significantly increases the abrasion resistance of the surface layer. Coatings are used to increase corrosion protection and tribological properties [5,6,7]. Sahoo and Suman [8] reviewed the tribological advancement of different electroless nickel coatings depending on the structure and testing parameters. Nanocomposite coatings consist of ceramic, polymer particles or nanoparticles (SiC, Si_3_N_4_, Al_2_O_3_, PTFE, diamond, etc.) in a metallic matrix made of a nickel–phosphorus alloy. Coatings with ceramic particles show a high hardness and abrasion resistance, which influences the mechanical and tribological properties of the coatings. However, too high a content of nanoparticles reduces the mechanical and tribological properties. It is essential to use an appropriate method of producing coatings. According to the results of the research conducted by Varshney et al. [9], the material underwent an increase in microhardness of 14% following the grain boundary relaxation heat treatment. Vijayanand and Elansezhian [10] found that it improved the wear resistance from ∼30 μm to ∼−70 μm and that the average coefficient of friction of the AHL samples decreased from 0.079 to 0.009. Similarly, the studies by Touri and Monirvaghefz [11] showed that heat treatment causes a significant increase in hardness in the section containing phosphorus, which radically increases its wear resistance. According to Kanamori et al. [12], owing to the post-heat treatment, it was found that the hardness of the coating, adhesion strength and durability were improved. Nanoparticles improve the properties of the coatings, but only the optimal selection of the dispersion phase, particle size and content in the coating will allow obtaining a layer with the required properties for applications in the expected operating conditions [13,14,15,16,17]. Szala and Kot [18] proved that repainting polyester powder coatings does not affect the microstructure and adhesion of the coating to the substrate, the results of the bending tests and the roughness of matt and silky coatings. Overcoating has been found to affect both the results of impact and stamping tests and the roughness of samples with a fine surface finish. Preliminary results of tribological tests performed with the three-ball-cone and roller-disc method showed that the Ni-P/Si_3_N_4_ layers of Si_3_N_4_ nanoparticles on the AW-7075 aluminum alloy increase the mechanical and tribological properties compared to the Ni-P layer [19,20]. Moreover, preliminary results of tribological tests of Ni-P/Si_3_N_4_ layers deposited by the chemical reduction method showed that the coating is characterized by better mechanical and tribological properties than the AW-7075 alloy [21].

Based on the literature analyses carried out in the area of Ni-P/Si_3_N_4_ nanocomposite coatings, it was found that the tests so far were carried out with the content of the Si_3_N_4_ dispersion phase within the range of precisely 0.5–10 g/dm^3^. While comparing the publications, it was noticed that the research methods, material conditions, test conditions, times and other factors had a clear impact on the final results obtained. It was also noted that the improvement of the tribological properties of Ni-P/Si_3_N_4_ nanocomposite coatings is achieved depending on the test conditions; e.g., according to [20], in specific time ranges (for the Ni-P/Si_3_N_4_ nanocomposite coating produced in a bath of a content of 10 g/dm^3^ Si_3_N_4_), or, according to [22], only as a result of the application of heat treatment (for the Ni-P/Si_3_N_4_ nanocomposite coating produced in a bath with 0.5 g/dm^3^ Si_3_N_4_ content). However, the mechanical and tribological properties improve due to the content of the dispersion phase. This article decided to include particles in the range of 0.5–10 g/dm^3^ Si_3_N_4_ in the chemical bath; more specifically, 2 g/dm^3^ and 5 g/dm^3^ are not extreme, but intermediate values. Extremely large or minimal values are not optimal values; therefore, from the point of view of the goal, i.e., searching for optimal values (i.e., improving both the mechanical and tribological properties of the amorphous nanocomposite Ni-P/Si_3_N_4_ coating), it was found that the selected values are appropriate at this stage when searching for optimal values. In addition, the preliminary research results [21] show an improvement in the properties of the Ni-P/Si_3_N_4_ coating material with an amorphous structure, which was deposited in the bath with Si_3_N_4_ particles at the level of 2 and 5 g/dm^3^, compared to the AW-7075 aluminum alloy, as well as the ordinary nickel Ni-P coating.

On this basis, it was found that nanocomposite layers are good materials for tribological applications in the case of AW-7075 aluminum alloys, which are now more widely used in mechanical engineering. Although some literature reports [20] that Ni-P/Si_3_N_4_ coatings remain in the phase of laboratory tests, the use of these coatings on an AW-7075 alloy is not yet recognized. The aim of this study was focused on identifying the basic mechanical properties of the coating. The conducted studies involved surface morphology and topography measurements, microhardness tests, adhesion analyses and friction coefficient determination. Moreover, the coating wear mechanism was identified based on tribological tests. Additionally, surface roughness tests were performed in order to determine the influence of Si_3_N_4_ nanoparticles on the friction coefficient parameter in tribological tests.

## 2. Materials and Methods

### 2.1. Plan of the Experiment

**Figure 1:**
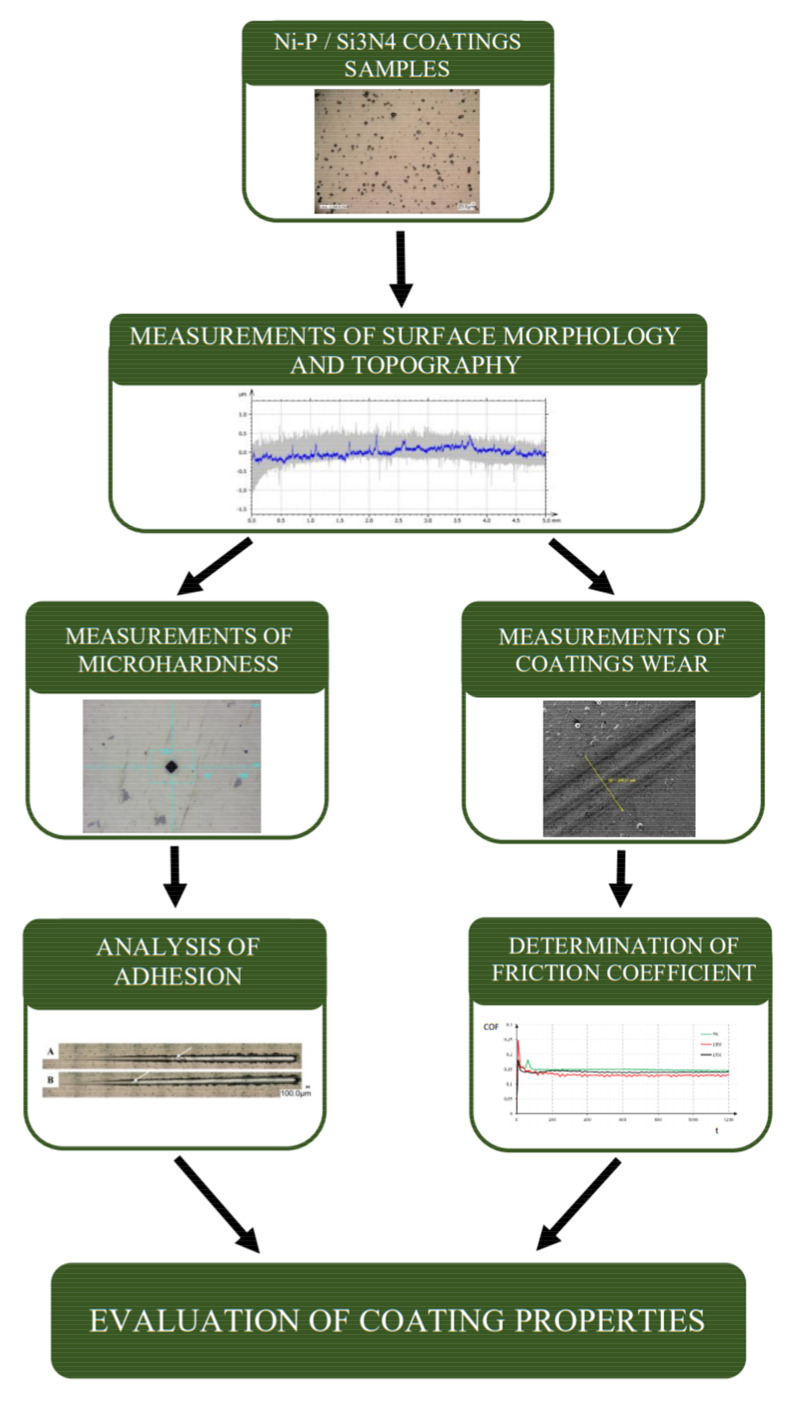
The scheme of a conducted experiment.

The basic mechanical parameters of Ni-P/Si_3_N_4_ nanocomposite coatings were evaluated as part of this study. Measurements of coating wear and evaluation of surface texture were performed during the experiments. Therefore, the microhardness and adhesion measurements have been applied. The tests were supplemented by the performance of complete tribological tests, including wear analysis and determination of the friction coefficient. Figure 1 presents the overall scheme of the conducted experiments.

### 2.2. Materials

The laboratory tests included Ni-P/Si_3_N_4_ coatings with different disperse phases deposited on the AW-7075 alloy by chemical reduction. The chemical composition of the AW-7075 alloy is presented in Table 1.

For the tests of hardness, morphology, roughness, adhesion and tribology, samples made of the AW-7075 alloy with dimensions D = 50 mm in diameter and g = 7 mm thick were used. Before depositing the Ni-P layers, the surfaces of the samples were degreased in an organic solvent, etched in an alkaline solution and galvanized in a multi-component solution. However, for the production of Ni-P layers by the chemical reduction method, a multi-component bath was prepared with the following composition: NiSO_4_, a reducer (NaH_2_PO_2_) and a buffer (C_2_H_3_NaO_2_), which stabilizes the pH at the level of 4.3–4.6. The bath temperature during the deposition process was 363 K. The nanocomposite layers were obtained by introducing the Si_3_N_4_ polydisperse powder in the form of a dispersion phase with a particle size of 20–25 nm. The thicknesses of the obtained coatings were the same and amounted to 10 ± 2 µm, while the Si_3_N_4_ phase content in the chemical bath was 2 g/dm^3^ and 5 g/dm^3^.

The Ni-P nickel and Ni-P/Si_3_N_4_ nanocomposite coatings were electroless deposited on the AW-7075 aluminium alloy, a disk-shaped substrate with a thickness of 7 mm and a diameter of 50 mm. The chemical composition of the alloy is given in Table 1. Silicon nitride Si_3_N_4_ was the dispersion phase in the nanocomposite coatings. Nickel sulfate, monosol phosphate (I) and hydroxy propionic acid were used in the bath.

The samples were degreased in acetone, etched in an alkaline solution (0.75% NaOH) (CHEMPUT, Piekary Śląskie, Poland) and galvanized in a multi-component solution; the composition and concentrations are given in Table 2.

For electroless deposition of the layers (by chemical reduction), a bath with a chemical composition was used, as shown in Table 3. A stabilized pH was obtained (pH 4.3–4.6). The bath temperature during the deposition process was approximately 363 K. The nanocomposite layers were obtained by introducing the polydisperse Si_3_N_4_ powder as a dispersion phase with a particle size of 20–25 nm. The content of Si_3_N_4_ nanoparticles in the baths was 2 g/L and 5 g/L. Ultrasonic mixing was also used before the deposition process and during the deposition of the layers, and consisted in mechanical agitation using a stirrer in the form of a glass rod with paddles in order to ensure proper dispersion of particles and to prevent sedimentation, as well as to obtain a homogeneous suspension and efficient transfer of the reinforcing phase. The thicknesses of all coatings were 10 ± 2 μm based on the selected deposition time, which was 60 min.

### 2.3. Surface Morphology and Topography Tests

**Figure 2:**
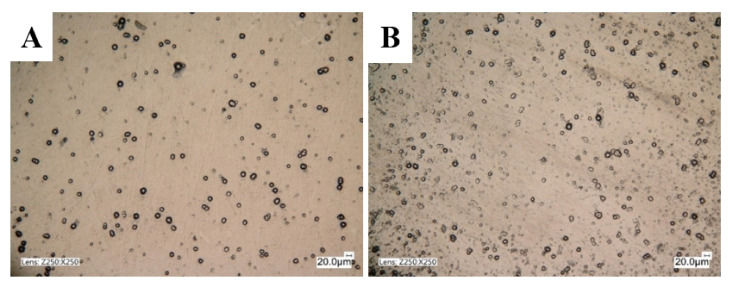
The morphology of the layers: Ni-P/Si_3_N_4_; (**A**) Ni-P/Si_3_N_4_ (2 g/dm^3^); (**B**) Ni-P/Si_3_N_4_ (5 g/dm^3^).

**Figure 3:**
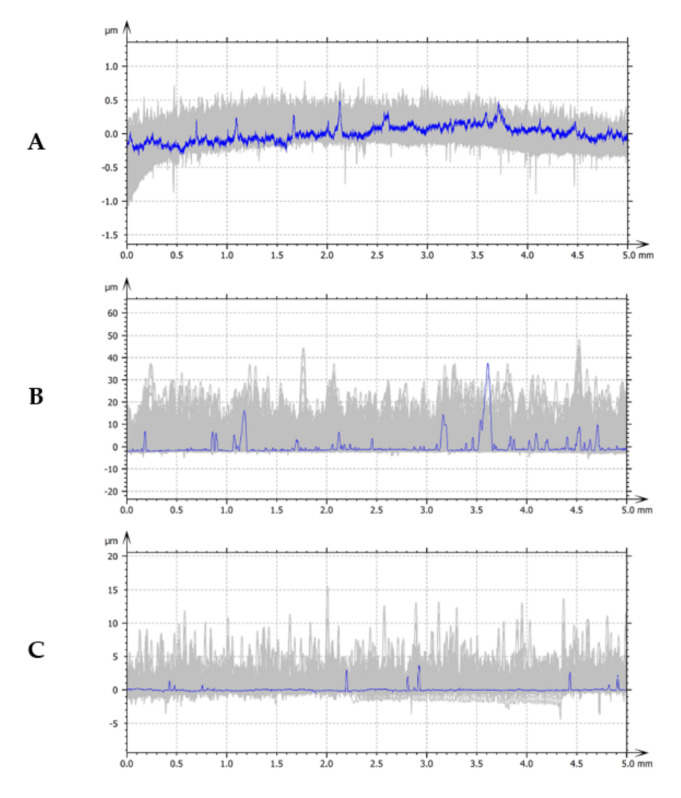
Topography of AW-7075 and Ni-P/Si_3_N_4_ coating surface: (**A**) AW-7075; (**B**) Ni-P/Si_3_N_4_ (2 g/dm^3^); (**C**) Ni-P/Si_3_N_4_ (5 g/dm^3^).

The surface morphology studies were carried out using the Keyence VHX 5000 optical microscope (Keyence Corporation of America, Itasca, IL, USA), while the surface topography was examined by scanning electron microscope Tescan Vega 5135 (Tescan Analytics, Fuveau, France) and profilometer Hommel ETAMIC T8000 (Jenoptik Industrial Metrology Germany GmbH, VS-Schwenningen, Germany) with the AltiMap Premium 7.1.7037 software (Jenoptik Industrial Metrology Germany GmbH, VS-Schwenningen, Germany). The topography tests were performed using the contact method in the middle parts of the samples. The measuring principle was to move the diamond measuring tip with a constant speed of 90° and a rounding radius from 2 µm over a precisely defined measuring section. The study of the surface topography revealed the parameters of the height of the peaks and roughness and the areas of depressions, elevations and bulges. Surface roughness tests were carried out individually, on each sample separately, and included a square of 5 mm × 5 mm surfaces in the central part of the sample. In turn, the morphology of each sample was repeatedly tested in several places. Figure 2 shows sample images, while the roughness test results are shown in Figure 3 and Table 2.

### 2.4. Microhardness and Adhesion Tests

The microhardness of the alloy system and the layered coating material was tested using the DSI (depth-sensing indentation) method on the PICODENTOR HM500 device (Helmut Fischer Gmbh, Sindelfingen, Germany). The measurements were made using the Vickers method with a load of 300 mN for 20 s and the holding of this force for 5 s. The microhardness test was carried out without piercing the material of the tested layers. The averaged measurement results (five measurements were made on each sample) are presented in Table 3. The tests of adhesion of coatings to the AW-7075 alloy were carried out using the scratch test method. The tests were carried out on the CSEM Revetest device (Anton Paar GmbH, Graz, Austria), using a Rockwell indenter and an increasing progressive load of 1 N to 100 N at a constant speed of 10 mm/min. The length of each scratch was 10 mm. Two scratches were made on each sample, and the acoustic emission signal, friction force, friction coefficient and normal force were recorded in parallel during the measurements.

### 2.5. Tribological Tests

The tribological properties of the layers deposited on the AW-7075 substrate were tested using the ball-on-flat method in reciprocating motion, in which the counter-sample was a bearing ball. The research was carried out with the use of the BRUKER UMT-2 Tribolab (Bruker Corporation, Billerica, MA, USA). The tribological tests were carried out in accordance with the applicable PN-EN 1071-12 standard. All measurements were carried out at various loads (5 N, 10 N, 15 N) using GL-4 75W/90 semi-synthetic gear oil. The samples were placed in a unique oil-filled tub, and then a counter-sample was mounted, which exerted appropriate pressure on the tested sample surface. Traces of wear were subjected to detailed microscopic analyses, which were made using the Keyence VHX 5000 optical microscope. The wear criterion was the width of the cracks formed according to the PN-EN 1071-12 standard. In addition, during the tests, changes in the friction coefficient were recorded, which, in combination with microscopic images, were used to determine the resistance of the layers to abrasive wear. The parameters are shown in Table 4.

After the measurements, the samples were again subjected to microscopic examination in order to thoroughly check the surface condition and width of the resulting scratches. Moreover, changes in the friction coefficient parameter recorded by the measuring machine were analyzed.

## 3. Results and Discussion

### 3.1. Evaluation of Coating Properties

The results of the morphological tests are shown in Figure 2. The matrix of the composite of layers produced was a solid solution of phosphorus in nickel Ni−P containing 6% P mass by the chemical reduction method. According to [5,13,23], the coating structure depends on the type of layer and composition. In a composite material, the structure is determined by constructing the matrix material, the type of dispersion ceramic phase, the degree of fragmentation and the material’s content. The content of the dispersion ceramic phase in the nanocomposite coating depends on the concentration of nanoparticles in the electroplating bath. As the Si_3_N_4_ content in the bath grows, the number of particles integrated into the deposited coating material increases, contributing to the formation of highly concentrated places.

The morphology results confirm that homogeneous and compact structures characterize all tested coatings. In the content of the dispersion phase, the nanocomposite layers are different from each other. The results of qualitative tests of the surface of Ni-P/Si_3_N_4_ layers deposited in the bath with 5 g/dm^3^ Si_3_N_4_ content showed an increased porosity and roughness compared to the layers deposited in the bath with 2 g/dm^3^ Si_3_N_4_ content. The reason is due to the more significant number of peaks, which was confirmed in later profilometric tests. The “dots” visible in Figure 2 are the peaks of the coating irregularities, the topography of which is shown in Figure 3. Increasing the concentration of Si_3_N_4_ causes an increase in the number of peaks.

**Figure 4:**
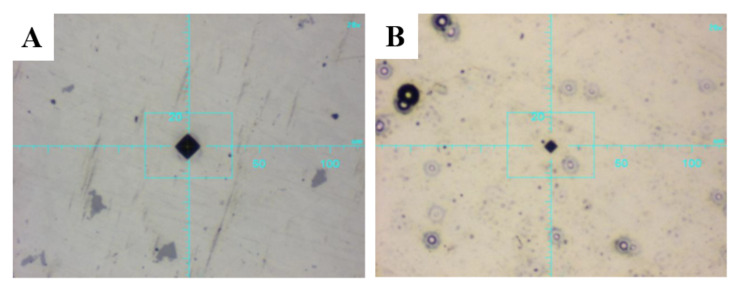
Images of traces on sample surfaces (magnification 20×; (**A**) AW-7075; (**B**) Ni-P/Si_3_N_4_).

The results presented in Table 5 and Figure 4 show two-dimensional images of the examined surfaces. In both cases, the surfaces are dull and devoid of gloss, and the peaks of the unevenness are even. Material defects, such as discontinuities, cavities or microcracks, were not observed.

### 3.2. Evaluation of Microhardness and Adhesion

The 10 µm thick Ni-P/Si_3_N_4_ coatings present a larger hardness than the AW-7075 aluminium alloy. The highest hardness characterizes coatings produced in a bath containing 2 g/dm^3^ of the dispersion phase. The content of Si_3_N_4_ nanoparticles in the coatings was different. Increasing the content of the dispersion phase to 5 g/dm^3^ reduces the hardness of the layer. This relationship was confirmed on all tested samples. The results are presented in Table 6 and Figure 4.

**Figure 5:**
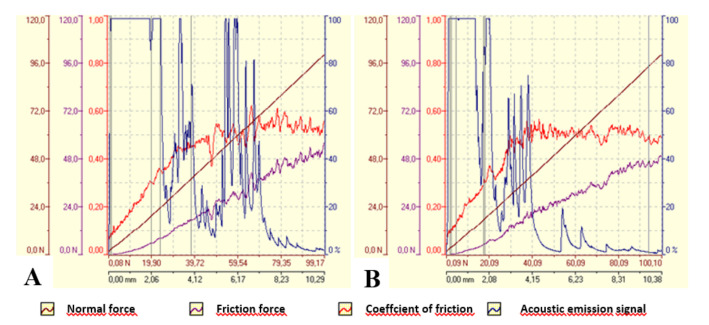
Scratch tests results for Ni-P/Si_3_N_4_ coatings: (**A**) Ni-P/Si_3_N_4_ (2 g/dm^3^); (**B**) Ni-P/Si_3_N_4_ (5 g/dm^3^).

**Figure 6:**
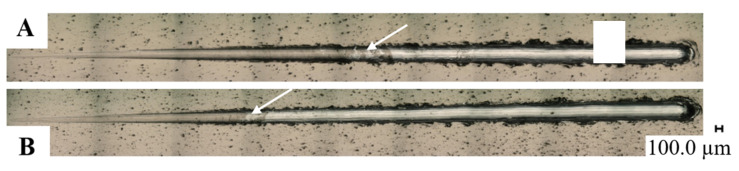
Images of scratches on the surfaces of layers: Ni-P/Si_3_N_4_; (**A**) Ni-P/Si_3_N_4_ (2 g/dm^3^); (**B**) Ni-P/Si_3_N_4_ (5 g/dm^3^). The white arrows indicate the coating destruction point.

A scratch test was used to analyze the strength of the bond between the coating and the substrate. The diamond indenter moved along the surface of the test layer at a constant speed with a constantly increasing loading force. Changes in individual parameters recorded during the test, i.e., normal pressure, friction force, friction coefficient and acoustic emission along the crack, are shown in Figure 5. The images of the cracks are shown in Figure 6.

However, detailed analyses of the results are presented in Table 7 and Table 8. Changes in the topography are noticeable at low-pressure values (0.76 N and 1.73 N), propagating, according to the load, transversely to the head movement.

In Table 7 and Table 8, the damages of a cohesive and adhesive character are marked with colors. It was difficult to determine at what load the first cracks began. The focus was on cracks that were not single and random, as well as damage to the coatings that began to appear and grow and had a clear and indisputable cohesive or adhesive character. Detailed values of the critical loads are presented in Table 9. For both coatings, the lower limit loads are in a similar range of 19.73N and 17.91N. An evident change is noticeable in the critical load, during which the coating breaks; the values differ significantly. The moment of coating decohesion is marked with a white arrow in Figure 6.

### 3.3. Evaluation of Tribological Parameters

**Figure 7:**
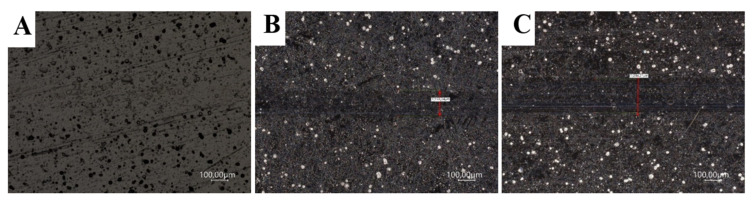
Images of scratches for samples with NiP/Si_3_N_4_ coatings deposited in a bath with 2 g/dm^3^ Si_3_N_4_ (load: (**A**) 5 N; (**B**) 10 N; (**C**) 15 N; magnification: 500×).

**Figure 8:**
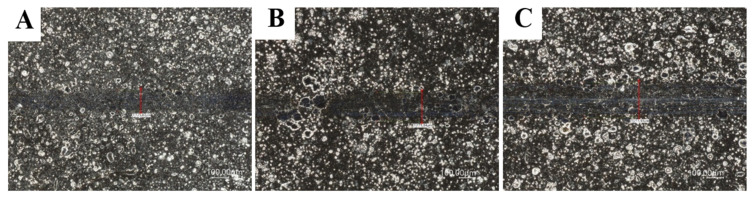
Images of cracks for samples with NiP/Si_3_N_4_ coatings deposited in a bath with 5 g/dm^3^ Si_3_N_4_ (load: (**A**) 5 N; (**B**) 10 N; (**C**) 15 N; magnification: 500×).

**Figure 9:**
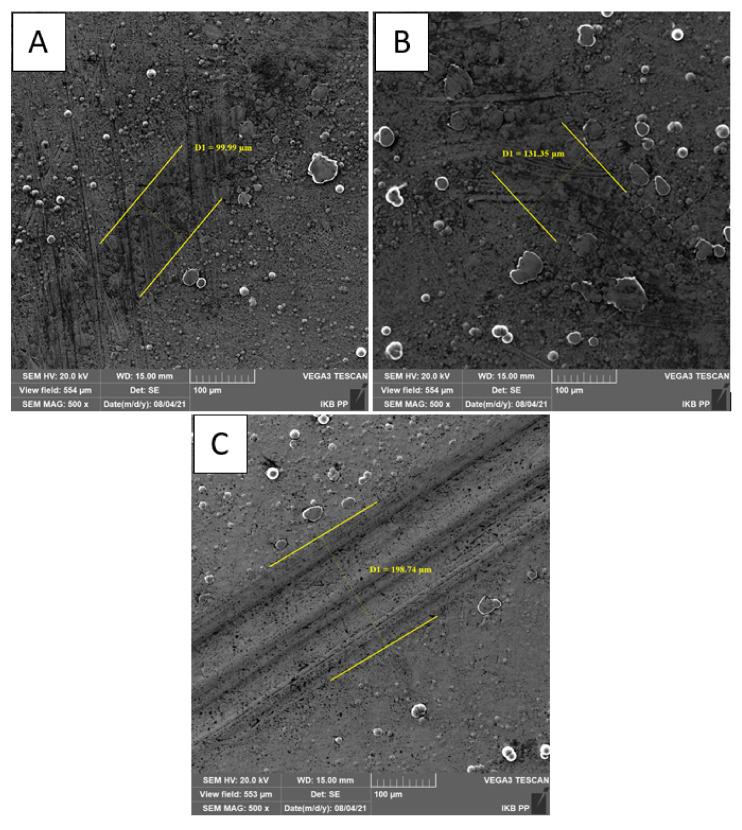
SEM Images of cracks for samples with NiP/Si_3_N_4_ coatings deposited in a bath with 5 g/dm^3^ Si_3_N_4_ (load: (**A**) 5 N; (**B**) 10 N; (**C**) 15 N; magnification: 500×).

Figure 7, Figure 8 and Figure 9 show the results of tribological tests performed with the ball-on-flat method, which were carried out during 1200 s using GL-4 75W/90 semi-synthetic gear oil. All other parameters and wear diameters remained unchanged. Specialized computer software was used again, which is an integral part of the light microscope. The drawings show high-resolution microscopic images showing the width of the carved grooves to an accuracy of 0.1 µm.

Based on the results of the second tribological tests stage, which were carried out on all samples, it was found that Ni-P/Si_3_N_4_ (2 g) coatings both contribute to the reduction of surface wear and are resistant to damage. The value of the loading force had the most significant impact on the size of the cracks. The content of the dispersion phase increases the abrasion resistance of the surface layer, but increasing its content from 2 to 5 g/dm^3^ in the chemical bath resulted in a slight increase in surface wear, as the crack width slightly raised to the range of 138.7 ÷ 152.5 µm. The lowest wear of the coating surface was recorded for the sample with the Ni-P/Si_3_N_4_ (2 g) layer, on which the crack widths were imperceptible and disappearing; therefore, their width cannot be precisely determined in this case. The exact results of the width of the resulting cracks are presented in Table 10.

The microscopic images of the resulting cracks, shown in Figure 7 and Figure 8, clearly differ from each other. The crack widths characterizing the degree of surface wear depended on the pressure force of the counter-sample and the type of the tested surface, the use of lubricant and the duration of the tests at a given speed. Crack widths are not the only determinant of surface wear and its behavior under given operating conditions. The sample with the applied layer of Ni-P/Si_3_N_4_ (2 g) nanocomposite showed the highest resistance to any signs of wear because, during microscopic observations, it is difficult to see traces of the counter-sample for the 5 N force. In Figure 7a, no signs of wear have been noticed. The Ni-P/Si_3_N_4_ (5 g) layer showed higher wear than the previous sample because the scratches were distinct and larger than the Ni-P/Si_3_N_4_ (2 g) layer and its width was within the range of 114.0 ÷ 118.5 µm. It was noticed that these layers are characterized by a very high resistance to abrasion and puncturing of the coating.

Increasing the pressure of the counter-sample from 5 to 10 N, and then to 15 N, resulted in more expansive and distinct cracks; the edges and abrasion marks of the counter-sample passage are more visible. The SEM photos and the measurements of the width of the paths show that the 15 N force caused the most visible deformation of the surface of the samples (see Figure 9). The analysis of the cross-section profiles of the tracks using SEM did not show any significant differences between the samples. Similarly, no damage to the surface of the coatings in the form of cracks, delamination and material defects or nicks was found. In addition, the influence of the content of the disperse phase on the tribological properties is more noticeable at lower loads. The research confirmed that nanocomposite layers deposited on the AW-7075 alloy can be subjected to loads of various values and can be successfully operated in multiple environments using lubricants. It was confirmed that the tested layers contributed to the increase in the surface resistance to tribological wear to a different extent. Table 10 summarizes the crack widths for the individual samples in order to more accurately illustrate the degree of wear.

**Figure 10:**
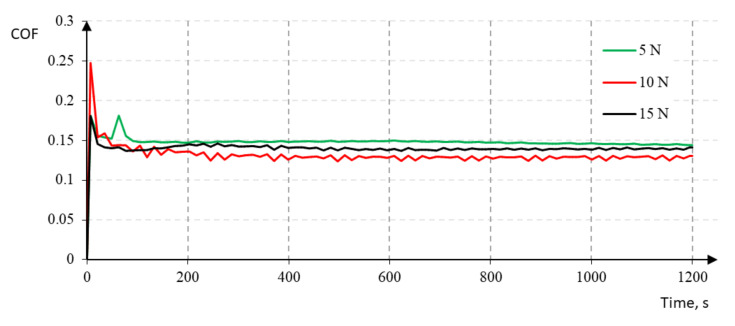
The friction coefficient measurements for Ni-P/Si_3_N_4_ (2 g) sample with different basic loads.

The example values of the measured friction coefficients were shown in Figure 10. The charts of changes in the friction coefficient were obtained automatically from tribological tests using the computer software attached to the BRUKER UMT TriboLab device. For all samples, the value of the friction coefficient was stabilized.

The obtained diagrams show that the friction coefficients for the Ni-P/Si_3_N_4_ (2 g) layers with a thickness of 10 μm stabilize after 40 s at one level in the range 0.12–0.16 (see Figure 10). On the other hand, nanocomposite Ni-P/Si_3_N_4_ (5 g) layers with an increased content of the dispersion phase are characterized by a similar, slightly higher coefficient, which oscillated in the range of 0.14–0.15, which is reflected in the results of profilometric tests that confirmed a greater roughness. Moreover, the surfaces of the Ni-P/Si_3_N_4_ (2 g) nanocomposite layers showed significantly higher abrasive wear resistance—for the 5 N load, the crack was imperceptible, while for the 10 N and 15 N loads, the crack widths are much smaller compared to the Ni-P/Si_3_N_4_ layer (5 g), where the scratches are clearer and appear regardless of the load.

## 4. Discussion

The aim of this study was focused on identifying the basic mechanical properties of the coating. The obtained results provide the first instance in literature of essential information regarding the Ni-P/Si_3_N_4_ coatings’ mechanical properties, and enable the selection of a practical application of these coatings. These results are the basis for further research on using Al-Zn-Mg alloys with deposited nickel layers in the engineering industry. The development of the work is both to increase the durability of various materials used for machine parts and the possibility of improving the efficiency of subassemblies by reducing the weight of moving parts subject to loads.

Analyzing the literature, it can be easily seen that the problems related to Si_3_N_4_ and NI-P are very extensively analyzed. Mazurek et al. [24] showed that Ni-B/Si_3_N_4_ composite layers and Ni-B composite layers are characterized by compact structures and a good adhesion to the substrate material. The incorporation of Si_3_N_4_ particles into the Ni-B layers increases the degree of surface development of the layers. The Ni-B/Si_3_N_4_ composite layer material exhibits less microhardness and less abrasive wear compared to Ni-B layers. However, the extent of wear damage of the Ni-B/Si_3_N_4_ is relatively small when compared to the Ni-B layers. Liua et al. [25] studied the impact of Si_3_N_4_nws to CF-HA/resin composites. The tensile strength of the CF-Si_3_N_4_nws-HA/resin composites increased by 41.7% compared with CF-HA/resin composites. The wear rate decreased by 85.8% compared with CF-HA/resin composites. CF-Si_3_N_4_nws-HA/resin composites showed good in-vitro bioactivity and have the potential for applications in bone graft. Cao et al. [26] noticed that relatively porous Si_3_N4 ceramics with special three-dimensional cage structures have a high mechanical strength, good antioxidation properties and excellent dielectric properties. According to Khullar et al. [27], the electrochemical, mechanical and microscopic inspection data supported the hypothesis that the Si_3_N_4_/Ti-6Al-4Vcombination had a better fretting corrosion performance compared to CoCrMo/Ti-6Al-4V. Zhanga et al. [28] compared the the friction coefficient of the Si_3_N_4_/stainless and Si_3_N_4_/PEEK sliding pair with an increasing load of 10 N to 30 N. For the first pair, the friction coefficient increased from 0.48 to 0.72, whereas for the second pair, it decreased from 0.27 to 0.07. However, the wear rates of these two sliding pairs were consistent.

Trzaska et al. [29] proved in his research that the incorporation of the PTFE powder particles in the nickel–phosphorus matrix increases the degree of surface development and hardness of the coating material and increases the wear resistance. Li et al. [30] noticed that, compared to the as-deposited Ni-P coatings, the toughness of the annealed composite coatings improved significantly. The superelastic effect of NiTi particles was observed in the Hertzian indentation behavior of the annealed composite coatings. In the study, Mohsenifar and Ebrahimifar [31] studied properties of the Ni–P–Al_2_O_3_–TiO_2_ composite coating, which was made on AISI 316 steel using a direct current deposition technique. The microstructure of the coating and its corrosion resistance were studied by changing the amount of titanium oxide (1, 2, 3 and 4 g/L) in the bath. The results of these tests were also correlated with microscopic images and showed that the coatings in a bath containing 4 g/L titanium oxide have the highest corrosion resistance.

## 5. Conclusions

The tested Ni-P/Si_3_N_4_ nanocomposite coatings, with a thickness of 10 μm, were deposited on the AW-7075 aluminum alloy. The results show that nanocomposite coatings have better mechanical and tribological properties than the AW-7075 alloy. Moreover, the influence of the content of the dispersion phase on the tested properties is noticeable. Ni-P/Si_3_N_4_ nanocomposite layers produced in a 2 g/dm^3^ bath exhibit different mechanical and tribological properties than coatings produced in a 5 g/dm^3^ bath, especially hardness and wear resistance. The best results were obtained for coatings produced with a dispersion phase with a content of 2 g/dm^3^ in the bath during deposition. Based on the carried out test, the following conclusions were formulated.

- It is noteworthy that despite the largest observed values of the roughness parameters of Ni-P/Si_3_N_4_ nanocomposite layers (2 g/dm^3^), their surfaces also show the highest resistance to abrasive wear;
- Increasing the content of the dispersion phase to 5 g/dm^3^ resulted in a bit decrease in hardness and wear resistance;
- The results of the scratch adhesion test showed that adhesive cracks begin to appear at various loads and are dependent on the thickness of the chemical composition of the coatings, and, more precisely, on the content of the dispersion phase;
- Generally, the Ni-P/Si_3_N_4_ layers are characterized by good bonding to the base material, especially coatings deposited in a bath with a content of 2 g/dm^3^. In the case of this coating, both adhesive and cohesive cracks appear much later at higher loads compared to the Ni-P/Si_3_N_4_ coating produced in a bath with a content of 5 g/dm^3^;
- Both the results in the form of microscopic images and measured numerical values prove the advantage of the Ni-P/Si_3_N_4_ coating obtained in the bath with the content of 2 g/dm^3^ in terms of its adhesion to the aluminum substrate compared to the Ni-P/Si_3_N_4_ coating produced in a bath with a content of 5 g/dm^3^;
- The results obtained in the study prove that the Ni-P/Si_3_N_4_ nanocomposite coating–AW-7075 substrate system is a good areological system;
- The tests also confirmed that the tested nanocomposite layers are promising materials for further mechanical and tribological tests. The coatings were deposited on polished and smooth substrates; therefore, in order to improve the surface properties, the polishing process should be repeated after creating the layers.

## Figures and Tables

**Table 1:** Chemical composition of AW-7075 alloy, % by mass.

Chemical Composition [%]										
Zn	Mg	Cu	Fe	Si	Mn	Cr	Zr	Ti	Rest	Al
5.1–6.1	2.1–2.9	1.2–2.0	max 0.50	max 0.4	max 0.3	0.18–0.28	max 0.25	max 0.20	max 0.05	other

**Table 2:** Components concentrations of multi-constituent substance for galvanizing.

Substrate	Chemical Formula	Concentration [g/dm^3^]
Sodium hydroxide	NaOH	120
Zinc oxide	ZnO	12
Nickel (II) sulfate	NiSO_4_·6H_2_O	1.5
Iron (III) chloride	FeCl_3_·6H_2_O	2
Sodium potassium tartrate	KNaC_4_H_4_O_6_·4H_2_O	15
Sodium citrate	C_6_H_5_O_7_Na_3_·H_2_O	15

**Table 3:** Components concentrations of nickel deposition bath.

Substrate	Chemical Formula	Concentration [g/dm^3^]
Monosodium phosphate (I) (reducer)	NaH_2_PO_2_·H_2_O	30
Sodium acetate	CH_3_COONa·3H_2_O	35
Nickel (II) sulfate	NiSO_4_·6H_2_O	28
Lactic acid (pH stabilizing buffer)	C_2_H_4_OHCOOH	20

**Table 4:** Parameters of tribological tests.

Stage	The First	The Second	The Third
Load [N]	5	10	15
Time [s]	1200		
Length of wear track [mm]	20		
Speed [mm/s]	2		
Counterspecimen	100Cr_6_ bearing steel ball with a diameter of 6.3 mm (1/4″)		

**Table 5:** Surface roughness test results.

Material	Thickness of Layer [µm]	Si Content in Layer [% vol.]	Si_3_N_4_ Content in Bath [g/dm^3^]	Rp ± SD [μm]	Rv ± SD [μm]	Rq ± SD [μm]	Rt ± SD [μm]
AW-7075	-	-	-	0.254 ± 0.036	0.168 ± 0.023	0.057 ± 0.005	0.584 ± 0.111
Ni-P/ Si_3_N_4_	10	0.44 ÷ 0.48	2	11.3 ± 2.68	2.44 ± 0.753	2.50 ± 0.696	23.9 ± 6.54
	10	0.58	5	2.30 ± 0.960	0.526 ± 0.153	0.337 ± 0.144	5.97 ± 2.33

**Table 6:** The coatings microhardness.

Material	The Thickness of Layer [µm]	Si_3_N_4_ Content in Bath [g/dm^3^]	HV0.03 ± SD	HM ± SD
AW-7075	-	-	202.62 ± 2.79	1643.46 ± 22.63
Ni-P/ Si_3_N_4_	10	2	642.47 ± 14.35	4306.35 ± 86.12
	10	5	638.70 ± 13.29	4283.43 ± 69.17

**Table 7:** Description of the scratch test for Ni-P/Si_3_N_4_ (2 g/dm^3^) layer.

Load [N]	Distance “x” [mm]	Description	Type of Failure
<0.76	<0.007	From the beginning of the scratch—longitudinal cracks at the outer edge of the scratch.	-
0.76	0.007	Lateral crack, possibly caused by points (dots) on the specimen, single perforation.	Cohesive
1.43	0.14	Longitudinal crack.	-
19.73	2.04	Cracks protruding from the outer edges of the scratch, material bulges at the outer edges of the scratch.	Adhesive
23.87	2.47	Beginning of small transverse cracks.	Cohesive
37.93	3.97	Larger single transverse cracks.	Cohesive
46.80	4.85	Layer perforation.	Adhesive
52.48	5.44	Larger crack with chipping.	Adhesive
54.60	5.66	Large layer perforation.	Adhesive
65.96	6.84	Breakage and destruction of the layer.	Adhesive

**Table 8:** Description of the scratch test for Ni-P/Si_3_N_4_ (5 g/dm^3^) layer.

Load [N]	Distance “x” [mm]	Description	Type of Failure
<1.73	<0.17	Minor longitudinal and transverse cracks with perforation caused by agglomerates passing through the visible surface of the sample.	Cohesive
1.73	0.17	Longitudinal cracks.	-
2.21	0.22	Longitudinal crack beyond the outer edge of the scratch.	-
4.43	0.45	Larger longitudinal fracture in the crack.	-
17.91	1.85	Beginning of growing cracks extending from the outer scratch edges.	Adhesive
28.96	3.00	Larger transverse crack in the crack.	Cohesive
33.22	3.44	Longitudinal break in a crack (larger).	-
34.95	3.62	Large cohesive fracture.	Cohesive
37.07	3.84	Layer perforation.	Adhesive
38.90	4.03	Breakage and destruction of the layer.	Adhesive
39.77 ÷ 43.91	4.12 ÷ 4.55	The highly perforated and cracked layer reappears, chipping, single exfoliation in the substrate.	-

**Table 9:** Critical loads for the tested chemical coatings.

Coating	Critical Load [N]		Coating Decrease [mm]
	L_c1_	L_c2_	
Ni-P/Si_3_N_4_ (2 g/dm^3^)	37.93	19.73	6.84
Ni-P/Si_3_N_4_ (5 g/dm^3^)	28.96	17.91	4.03

**Table 10:** Crack widths after tribological tests.

Material	AW-7075	Ni-P/Si_3_N_4_ (2 g/dm^3^)	Ni-P/Si_3_N_4_ (5 g/dm^3^)	AW-7075	Ni-P/Si_3_N_4_ (2 g/dm^3^)	Ni-P/Si_3_N_4_ (5 g/dm^3^)	AW-7075	Ni-P/Si_3_N_4_ (2 g/dm^3^)	Ni-P/Si_3_N_4_ (5 g/dm^3^)
Force [N]	5	5	5	10	10	10	15	15	15
Crack widths [µm]	129–133	-	116–126	165	159	150–175	210	196–204	196–211
The average value of crack widths [µm]	131	-	121	165	159	162	210	200	204

## Data Availability

The data presented in this study are available on request from the corresponding author. The data are not publicly available due to due to the large amount of data.
